# Regulation of Translation Initiation under Biotic and Abiotic Stresses

**DOI:** 10.3390/ijms14034670

**Published:** 2013-02-26

**Authors:** Sira Echevarría-Zomeño, Emilio Yángüez, Nuria Fernández-Bautista, Ana B. Castro-Sanz, Alejandro Ferrando, M. Mar Castellano

**Affiliations:** 1 Centro de Biotecnología y Genómica de Plantas, INIA-UPM, Campus de Montegancedo, 28223 Madrid, Spain; E-Mails: sira.echevarria@upm.es (S.E.-Z.); yanguez.emilio@inia.es (E.Y.); nuria.fernandez@upm.es (N.F.-B.); anahermione@hotmail.com (A.C.-S.); 2 Instituto de Biología Molecular y Celular de Plantas CSIC-Universidad Politécnica de Valencia, Valencia, Spain; E-Mail: aferrando@ibmcp.upv.es

**Keywords:** regulation of translation, eIF4E, eIF2α, plant abiotic stress, IRES, cIRES, CITES, cap-dependent enhancers

## Abstract

Plants have developed versatile strategies to deal with the great variety of challenging conditions they are exposed to. Among them, the regulation of translation is a common target to finely modulate gene expression both under biotic and abiotic stress situations. Upon environmental challenges, translation is regulated to reduce the consumption of energy and to selectively synthesize proteins involved in the proper establishment of the tolerance response. In the case of viral infections, the situation is more complex, as viruses have evolved unconventional mechanisms to regulate translation in order to ensure the production of the viral encoded proteins using the plant machinery. Although the final purpose is different, in some cases, both plants and viruses share common mechanisms to modulate translation. In others, the mechanisms leading to the control of translation are viral- or stress-specific. In this paper, we review the different mechanisms involved in the regulation of translation initiation under virus infection and under environmental stress in plants. In addition, we describe the main features within the viral RNAs and the cellular mRNAs that promote their selective translation in plants undergoing biotic and abiotic stress situations.

## 1. Introduction

One key strategy for plants to adapt to the challenges imposed by biotic and abiotic threats is the modulation of gene expression. Changes of gene expression rely on the regulation of a wide variety of molecular mechanisms including mRNA transcription, processing, transport, translation, storage and decay. Among them, translational modulation emerges as a key step, since it is addressed to control the last step of protein production, it is reversible, and is exquisitely regulated [1,2]. Biotic and abiotic threats occur often suddenly and, therefore, a quick response is crucial to assure cell survival. In such a context, translational regulation of pre-existing mRNAs provides a fast and efficient way to control gene expression, especially when compared to the *de novo* mRNA transcription, processing and transport to cytoplasm [3].

Translation is a highly energy-demanding process and, consequently, it is one of the main targets to be inhibited in response to most types of cellular stresses. However, under conditions where global protein synthesis is severely impaired, some proteins mainly involved in homeostasis maintenance remain being synthesised as part of the mechanisms of cell survival. In the case of biotic stresses, this situation becomes more complex, as plant pathogens have also developed a plethora of sophisticated molecular mechanisms directed to efficiently synthesize the proteins encoded in their genomes ensuring their propagation.

In other eukaryotes, many examples of global translational inhibition and preferential production of key proteins that are critical for the adaptation to environmental threats are known [4–9]. Additionally, the molecular mechanisms by which animal pathogens are able to replicate, even when the translational machinery of the host-cell is blocked, have been broadly characterized [10]. Similar scenarios have also begun to be enlightened in plants, where several studies have demonstrated that the modulation of mRNA translation is a key point in the adaptation of plants to different challenging conditions [11]. In this review, we summarize the current knowledge on the translational control in plants in response to both abiotic and biotic stresses, focusing on the initiation step, which is the most finely regulated. In addition, we analyse the wide variety of strategies adopted by pathogens and plants to efficiently initiate the translation of their RNAs counteracting the restrictions imposed in each particular situation.

## 2. Regulation of Translation Initiation in Response to Stress Conditions

Protein synthesis is a key step of gene expression and it is specially regulated at the initiation phase. In eukaryotes, canonical cap-dependent translation begins with the recognition of the mRNAs 5′-cap structure (7-methyl-guanosine) by eIF4E. The subsequent interaction of eIF4E with eIF4G and eIF4A allows the formation of the cap-binding complex, called eIF4F. Once eIF4F is formed, eIF4B and the pre-initiation complex 43S, which consists on the small ribosomal subunit 40S, the ternary complex eIF2/GTP/tRNA_i_^met^, and the factors eIF3, eIF1 and eIF1A, are recruited. Circularization of mRNA is afforded by interaction between the poly(A) binding proteins (PABPs) and eIF4G and eIF4B. Then, the 43S pre-initiation complex scans the mRNAs in the 5′–3′ direction until an initiation codon is found. At that point, the ribosomal subunit 60S is loaded, and the elongation phase begins [12].

Under a great number of threats, the eukaryotic cell reacts performing a drastic inhibition of translation initiation [2]. In mammals and yeast, some of the best characterized mechanisms of translation initiation repression affect the activity of the initiation factors eIF2 and eIF4E [13,14]. Interestingly, as mentioned above, a few mRNAs, frequently related to the stress response, are able to bypass this general repression and are efficiently translated [15]. In plants, however, the mechanisms of translation inhibition under stress conditions are mainly unknown, and the processes by which some mRNAs are selectively translated remain to be elucidated [16]. All these general mechanisms, which modulate the efficiency of the initiation step, are analysed in detail below.

### 2.1. Regulation of eIF2α Activity in Response to Stress

In eukaryotes, the regulation of eIF2 is mediated by the phosphorylation of its α subunit by selective eIF2α kinases. The phosphorylation of eIF2α prevents the formation of the eIF2/GTP/tRNA_i_^met^ ternary complex, resulting in a strong inhibition of translation [14]. However, in this general inhibition scenario, two mRNAs, *GCN4* and *ATF4*, whose translation is not affected by eIF2α phosphorylation, have been characterized in yeast [17] and mammals [18], respectively. Recently, the selective translation of different transcription factors, zinc-finger and bromodomain-containing proteins, and proteins related to cell cycle, to cellular component movement and to stress response, have been described in mammalian cells under endoplasmic reticulum stress conditions, in which translation initiation is severely impaired by eIF2α phosphorylation [19].

Regarding biotic stresses, the best characterized example of eIF2α inhibition is observed in the infection by animal viruses. In this case, the activity of the ternary complex is mainly regulated by the PKR eIF2α kinase. This antiviral response causes an inhibition of cap-dependent translation initiation, addressed to avoid the production of new viral particles [20]. In turn, animal viruses have evolved different strategies to reduce eIF2α phosphorylation and to preserve the activity of the ribosomal machinery, trying to ensure the viral protein accumulation and the viral replication [21–23]. In plants, the participation of eIF2α phosphorylation as a mechanism of viral defence is still unclear. Indeed, phosphorylation of eIF2α is not observed in plants infected by, for instance, Turnip yellow mosaic virus (TuMV) or Turnip crinkle virus (TCV), pointing out that phosphorylation of this factor is not a part of the general plant response to the infection [24]. Moreover, the existence of a PKR-like kinase in plants remains controversial. On the one hand, genome-wide identification of eIF2α kinases in Arabidopsis and rice suggests the absence of a mammalian PKR-homolog protein [25]. On the other hand, silencing of the putative plant ortholog of the mammalian PKR inhibitor, p58^IPK^, leads to host death in *Nicotiana benthamiana* and Arabidopsis infected plants. This death is associated with phosphorylation of eIF2α in the case of *N. benthamiana*[26]. In addition, the expression of a non phosphorylable mutant version of eIF2α (S51A) in the described *N. benthamiana* silenced plants rescues the host from the death induced by the viruses [26]. Based on these data, neither the presence nor the absence of a plant PKR can be ruled out [24]. Further studies are needed to elucidate whether phosphorylation of eIF2α takes part in the translational response of plants to viral infection and how this phosphorylation can be achieved.

To date, only one eIF2α kinase, GCN2, has been found in plants [24,25]. Arabidopsis GCN2 is activated under different abiotic stress conditions, including amino acid and purine deprivation, cadmium, UV, cold shock and wounding [24,25]. However, the inhibition of translation induced by abiotic stresses in plants is not always accompanied by GCN2 mediated phosphorylation of eIF2α. This is the case of the stresses caused by NaCl, H_2_O_2_[25] and heat shock [27] in wheat. Interestingly, unlike mammals and yeast, no cases of mRNAs escaping eIF2α regulation have been found in plants so far.

### 2.2. Regulation of the eIF4E Activity under Stress Situations

The regulation of eIF4E under abiotic stress conditions is, by far, the best studied mechanism in other eukaryotes. One of the most interesting aspects of eIF4E regulation in mammals and yeast is its sequestration by the eIF4E-binding proteins (4E-BPs). In these organisms, under different stress situations, the kinase target of rapamycin (TOR) is inhibited, leading to the dephosphorylation of the 4E-BPs. Non-phosphorylated 4E-BPs interact with eIF4E, preventing the eIF4E-eIF4G conjunction and the subsequent recruitment of the ribosome to the mRNA cap structure. This process generates a switch in the translation from cap-dependent to cap-independent [13]. This mechanism has been also proposed to contribute to the inhibition of cap-dependent translation in viral infections [28]. In turn, animal viruses have also evolved molecular strategies to avoid 4E-BPs activation and to ensure viral protein synthesis and particle production [29].

In contrast to the situation in mammal virus infection, the regulation of the availability of the cap-binding complex (eIF4F) is unlikely to be involved in the plant antiviral defence. Indeed, no homologs of the 4E-BPs have been found in the plant genomes available to date. In addition, although two proteins, the β subunit of the nascent polypeptide associated complex (NAC) and the plant lipoxygenase 2 (AtLOX2), have been proposed to be the plant analogs of the mammalian 4E-BPs [30,31], no evidence for changes in translation mediated by these proteins has been described neither *in vitro* nor *in vivo.* Moreover, cap-dependent translation is unlikely to be completely inhibited upon infection, as some plant viruses synthesized capped mRNAs and the translation of other relies on an active eIF4F complex [32,33].

The involvement of this eIF4E controlling-pathway in the regulation of translation under abiotic stress situations is also unclear in plants. The Arabidopsis TOR kinase regulates protein synthesis and modulates tolerance to osmotic stress in the same direction as it does in mammals and yeast [34]. However, despite these parallelisms with other eukaryotes, the link between TOR, eIF4E regulation and cap-independent initiation under abiotic stress has not been so far elucidated in plants.

In mammals, a different regulation affecting eIF4E under stress conditions involves changes in its phosphorylation state. Dephosphorylation of mammalian eIF4E correlates with reduced eIF4F binding to the cap and protein synthesis activity [35]. Plant eIF4E has been found to be hyperphosphorylated under hypoxia [36] but not under heat stress [27]. However, the role of this phosphorylation on eIF4E activity in plants remains unresolved.

### 2.3. Other Mechanisms of Translation Initiation Regulation in Response to Stress

Beside these main regulators, other initiation factors, such as eIF1A, eIF4A and eIF3, seem to play an important role in the regulation of translation initiation under abiotic stress in plants. Sugar beet eIF1A confers salt tolerance when overexpressed in Arabidopsis [37]. In the same way, an eIF4A ortholog confers high salinity tolerance in tobacco [38]. Furthermore, eIF4A undergoes phosphorylation in response to hypoxia [39] and heat stress [27], a modification that has been considered a regulatory step inhibiting the 5′-cap recruitment or the unwinding of secondary structures of certain mRNAs [39].

Apart from the role of eIF3 in the canonical translation initiation, this factor is known to allow reinitiation after short open reading frames (ORFs) [40]. Recently, eIF3 has been found to play a key function in the reinitiation mechanism after long ORFs in Cauliflower mosaic virus (CaMV). During this process, eIF3 associates with translation reintiation viral and host factors, to form the TOR-regulated reinitiation-competent 40S complex, which is able to resume scanning and reinitiation of the downstream ORF [41].

Another layer of regulation, exclusive of plants, consists on the selective use of eIFiso4F to translate a specific set of mRNAs [42]. Interestingly, eIFiso4F allows a more efficient recruitment of mRNAs with higher 5′-UTR secondary structure and with hypermethylated cap structures [43]. In addition, Arabidopsis knockout mutants for both variants of eIFiso4G (eIFiso4G1 and eIFiso4G2) showed a similar rate of translation, but important phenotypic defects, suggesting that these isoforms are needed for the specific translation of physiologically relevant mRNAs [44]. Furthermore, in maize it has been demonstrated that eIFiso4E is particularly required for the translation of stored mRNAs from dry seeds, and that eIF4E is unable to fully replace this eIFiso4E function [45]. Whether this unique mechanism, regulating translation specificity in plants, plays a role in the selective recruitment of RNAs under stress conditions is an important question that should be further studied.

Beside the involvement of eIFs in translation regulation, another mechanism of control is that performed by micro RNAs (miRNAs). miRNAs are small RNA sequences able to hybridize with endogenous mRNAs by sequence complementarity, targeting those mRNAs for degradation or translational repression [46,47]. The single-stranded mature miRNAs associate with Argonaute (AGO) proteins to form the so-called RNA-induced silencing complex (RISC). This complex is then guided to the target mRNA, which can be just cleaved and degraded, or can be subjected to an interesting translational regulation mechanism known as miRNA non-cleavage repression. Although this repression is known to require mismatched complementarity between the miRNA and the target mRNA in mammals, this process has been recently proven to be functionally operative in plants despite near-perfect complementarity [48].

The role of miRNAs in abiotic stress response has been extensively described in plants [49–51]. However, reports of a non-cleavage translational regulation in the context of plant abiotic stress are hardly found [52], despite the fact that it is not so uncommon in developmental processes [53–55].

The molecular mechanism behind the translational regulation performed by miRNA is not clear, and whether it is directed to an inhibition in initiation, post-initiation, or both, is still under discussion [56]. In plants, some important observations have been made that can help to understand these mechanisms. Biochemical studies demonstrated that the presence of miRNAs and AGO1 in polysomes is correlated with translational repression in plants [57]. In addition, an implication for microtubules in miRNA translational regulation as well as a relationship with proteins of the processing bodies have been proposed [48]. Nevertheless, translational regulation by miRNA in plants is a novel research field and, thus, further investigation is needed to achieve a deeper knowledge of the molecular pathways involved in it.

## 3. Alternative Mechanisms of Translation Initiation in Virus and Plant mRNAs

Viruses do not possess the complex machinery required to translate their mRNAs, and are consequently forced to compete for and manipulate the host-cell translation apparatus to efficiently synthesize their proteins. To do so, plant viruses have evolved numerous unconventional mechanisms to recruit the cellular translational machinery to their viral messengers. These mechanisms can be classified according to their requirements for the cap structure into cap-dependent and cap-independent mechanisms. Examples of some of them have also been found in plant cellular mRNAs selectively translated in response to abiotic stresses. The different mechanisms, as well as their role in the efficient and selective translation of mRNAs under biotic and abiotic stress conditions are discussed below.

### 3.1. Non-Canonical Cap-Dependent Mechanisms

Many plant viruses have a positive single stranded genomic RNAs with a capped 5′-terminus but no poly(A) tail. These viruses overcome the lack of the poly(A)-tail by using a wide variety of structures at 3′-UTR that can functionally replace it. A representative example of this non-canonical translation initiation is observed in *Alfalfa mosaic virus* (AMV). AMV genomic RNA possesses a structured 3′-UTR that switches between two conformations: a pseudoknot which resembles a tRNA-like structure (TLS) and a coat protein binding site (CPS) [58]. The CPS conformation allows the binding of the coat protein (CP), which, in turns, interacts with eIF4G and eIFiso4G. This interaction promotes the circularization of the mRNA and the efficient recruitment of the whole translation machinery [32].

Other examples of cap-dependent enhancers are those found in *Potato virus X* (PVX) and in *Tobacco mosaic virus* (TMV). The PVX capped 5′-terminus contains an untranslated region that enhances ribosome binding by partial complementarity with 18S ribosomal RNA [59–61]. In the case of TMV, the efficient translation of its viral proteins relies on two translational enhancers: the omega (Ω) leader sequence and a highly conserved upstream pseudoknot domain (UPD) placed at the 3′-UTR [62,63]. The Ω leader, a capped 68 bp 5′-UTR sequence, facilitates the recruitment of eIF4G in an HSP101-dependent mechanism [63,64]. Interestingly, the UPD can also bind HSP101, suggesting a synergistic role for these two enhancers in TMV translation [32].

Although the role of these cap-dependent enhancers in plant virus translation is well established, to date, no cap-dependent enhancer involved in the selective translation of plant cellular mRNAs under abiotic stress has been described. However, their existence cannot be completely dismissed as this field has been poorly studied so far.

### 3.2. Cap-Independent Mechanisms

Plant viruses, such as the members of the *Potyviridae, Comoviridae, Tombusviridae* and *Luteoviridae* families, lack the 5′-cap structure. Consequently, these viruses have developed efficient cap-independent mechanisms of translation, addressed to ensure satisfactory levels of their encoded proteins in the host cells. These mechanisms range from the use of viral proteins that functionally replace the cap structure to the use of internal ribosome entry sites (IRESs), cap-independent regulatory elements (CIREs) and 3′-cap independent translation elements known as CITEs.

Examples of viruses that use a viral protein to recruit the translation machinery are the *Potyviridae*. In these viruses, the viral protein VPg is covalently linked to the 5′-terminus of the viral genomic RNA and interacts with eIF4E and eIFiso4E [65,66]. The interaction of the VPg with eIF4E and eIFiso4E is crucial for the viral cycle, as point mutations in the eIF4E isoforms result in resistance to potyviral infection in different plant species [67,68]. Interestingly, distinct potyviruses differ in the use of the different host eIF4E isoforms. This is found in Arabidopsis, where knockout mutants for eIFiso4E are resistant to TuMV, *Lettuce mosaic virus* (LMV) and *Tobacco etch potyvirus* (TEV), but not to *Clover yellow vein virus* (ClYVV) [69,70]. On the contrary, eIF4E knockouts prevent infection by ClYVV, but not by TuMV [71]. In pepper, mutation of an *eIF4E* gene confers resistance to *Potato virus Y* (PVY) and TEV, while resistance to *Pepper venial mottle virus* (PVMV) requires mutation for both *eIF4E* and *eIFiso4E* genes [72]. Moreover, potyvirus specificity for the different eIF4E isoforms depends also on the host, as LMV and TEV require eIFiso4E to replicate in Arabidopsis [69,70], while same strains need eIF4E to infect pepper, tomato and lettuce [73–75]. Although all these data highlight the relevance of the eIF4E isoforms in the virus cycle, the role of the interaction between VPgs and eIF4E isoforms in translation remains unclear, but if any, it may help to promote translation of the viral mRNAs [76] or may drive translation inhibition of cellular mRNA by sequestering eIF4E [77,78].

By far, the most studied mechanism leading to cap-independent translation of viral RNAs is the use of internal ribosomal entry sites (IRESs). IRESs have been found to drive cap-independent translation of viral RNAs in members of diverse plant virus families as *Potyviridae*, *Luteoviridae*, *Tobamoviridae* and *Tombusviridae*[79]. IRESs engage the ribosomal machinery and bring it directly to close proximity of the initiation codon. Although there is little similarity of sequence and structure among the different classes, all IRESs have evolved to allow efficient translation initiation independently of the key components of the translation initiation machinery. As it is also observed in mammals, the different IRESs of plant viruses function by very diverse mechanisms, with translation initiation factor requirements ranging from almost all canonical initiation factors, with the exception of eIF4E, to almost none [80]. However, IRES elements of plant viruses differ significantly from those found in animal viruses, as they are generally smaller (<200 nt), less structured and sometimes located outside the 5′-UTR.

A different group of elements that promote the cap-independent translation of viral proteins are the cap-independent regulatory elements or CIREs. A representative example of plant virus CIREs is found in TEV. The TEV 5′-leader sequence contains two CIREs: a 45 nt RNA pseudoknot (PK1) and an unstructured element that resembles the TMV Ω sequence. One loop in the PK1 shows complementarity to the 18S ribosomal RNA. Therefore, PK1 has been proposed to cooperate with the unstructured element to enhance translation of TEV by facilitating the recruitment of the 40S ribosome subunit directly to the viral RNA [81].

In addition to IRESs and CIREs, some RNA viruses have evolved efficient cap-independent strategies that involve sequences located in the 3′-UTRs. These sequences, generally known as CITEs, are only present in plant RNA viruses. As well as IRESs, CITEs show no apparent sequence or structural similarity. However, almost all share some features: (1) they contain complex RNA structures that generate functional bridges between the 5′and 3′-UTRs of the viral RNA by long-distance base pairing [82]; and (2) they promote the efficient recruitment of distinct components of the translation initiation machinery to the viral RNA. Thus, although viral CITEs promote cap-independent translation [83], some canonical initiation factors are still required. This is the case of the 3′-CITE of the Pea enation mosaic virus (PEMV). The PEMV-like translation element (PTE) consists of a T-shaped RNA secondary structure [84] that engages in a long-distance RNA-RNA interaction and binds eIF4E [85]. In contrast, in the case of the translation enhancer domain (TED) of the Satellite tobacco necrosis virus (STNV), the recruitment of the translation machinery is supposed to be achieved by interaction with eIF4G or eIFiso4G [86].

In plants, so far, the presence of IRESs has been proposed the only possible mechanism underlying cap-independent translation of cellular mRNAs under abiotic stress conditions. Indeed, two cellular IRESs (cIRESs) have been found within the 5′-leader sequences of plant mRNAs involved in the heat and hypoxia stress response. These mRNAs code for the maize heat shock protein 101 (HSP101) [45] and the maize alcohol dehydrogenase 1 (ADH1) [87]. These cIRESs seem to enhance specific translation in plants, as the 5′-leader of ADH1 was able to provide efficient translation of a reporter mRNA *in vivo* in *Nicotiana benthamiana* cells [87]. Although promising, the examples of known plant cIRESs are scarce and, therefore, the assumption of cIRESs as a general mechanism to enhance translation of specific cellular mRNAs under abiotic stress in plants remains to be elucidated.

In addition, although the existence of CITE-like elements is still considered exclusive of plant viral mRNAs, it would not be surprising if such elements are also discovered in cellular mRNAs involved in triggering the plant response to environmental insults. This will provide an alternative to cIRESs to drive the selective translation of mRNAs under the general translation inhibition imposed by abiotic stress conditions [16].

## 4. Conclusions

In plants, regulation of translation plays a fundamental role in the modulation of gene expression under biotic and abiotic stresses. To date, an incomplete but considerable knowledge of the mechanisms used by plant viruses for the efficient synthesis of their proteins has been obtained. In this sense, although much work remains to fully decipher how viruses gain access to the host translation machinery, a remarkable number of diverse viral RNA elements able to recruit host translation factors have been identified. In contrast, we are far away from understanding how plants reprogram their mRNA translation upon abiotic stress. Indeed, further studies to identify the factors involved in translation inhibition in response to environmental cues are needed. In addition, new insights into the mechanisms that allow some cellular mRNAs to bypass the global translational blockage imposed by abiotic stresses should be ascertained. Only by profound comprehension of how translation is regulated, will we get closer to uncovering how plants respond to environmental challenges.
